# Effects of Repeated, Long-Duration Hyperoxic Water Immersions on Neuromuscular Endurance in Well-Trained Males

**DOI:** 10.3389/fphys.2019.00858

**Published:** 2019-07-24

**Authors:** Christopher M. Myers, Jeong-Su Kim, Kevin K. McCully, John P. Florian

**Affiliations:** ^1^Department of Nutrition, Food and Exercise Sciences, Florida State University, Tallahassee, FL, United States; ^2^United States Navy Experimental Diving Unit, Panama City Beach, FL, United States; ^3^Department of Kinesiology, University of Georgia, Athens, GA, United States

**Keywords:** water immersion, hyperoxia, neuromuscular endurance, electromyography, near-infrared spectroscopy, muscle oxidative capacity

## Abstract

**Purpose:**

This study examined the effects of repeated long-duration hyperoxic water immersions (WIs) at 1.35 atmospheres absolute (ATA) on neuromuscular endurance performance. We hypothesized that over a 5-day period of consecutive, resting, long-duration hyperoxic WIs there would be a decrease to neuromuscular endurance performance and tissue oxygenation with the quadriceps muscle, but not with the forearm flexors.

**Methods:**

Thirteen well-trained, male subjects completed five consecutive 6-h resting WIs with 18-h surface intervals during the dive week while breathing 100% oxygen at 1.35 ATA. We assessed skeletal muscle endurance performance before and after each WI, and 24 and 72 h after the final WI. Muscular endurance assessments included 40% maximal handgrip endurance (MHE) and 50-repetition maximal isokinetic (IK) knee extensions. Near-infrared spectroscopy (NIRS) was used to measure muscle oxidative capacity (MOC) of the vastus lateralis and localized muscle tissue oxygenation of the vastus lateralis and flexor carpi radialis. Simultaneously, we measured brachioradialis neuromuscular activation by surface electromyography (SEMG).

**Results:**

MHE time-to-fatigue performance declined by 15% at WI 3 (*p* = 0.009) and by 17% on WI 5 (*p* = 0.002). Performance continued to decline by 22% at 24-h post-WI (*p* < 0.001) and by 12% on 72-h post-WI (*p* = 0.019). Fifty-repetition IK knee extension total work decreased by 5% (*p* = 0.002) on WI 3, and was further reduced by 7.5 and 12.3% (*p* = 0.032) at pre-WI 5 and 24-h post-WI, respectively. However, the rate of fatigue was 8 (*p* = 0.033) and 30% (*p* = 0.017) lower at WI 3 and 24-h post-WI when compared to WI 1, respectively, demonstrating the muscles were still fatigued from the previous hyperoxic WIs. We detected no significant limitations in oxygen off-loading kinetics during the exercise or MOC measurements.

**Conclusion:**

Repeated, resting, long-duration hyperoxic WIs caused significant reductions to muscular endurance but not to indirect measures of oxygen kinetics in load bearing and non-load bearing muscles.

## Introduction

Exposure to elevated hydrostatic pressure for extended periods of time (e.g., 6-h) for specialized diving causes alterations to human performance (Florian et al., 2013; Myers et al., 2018a,b). These changes may adversely influence physical performance during the dive or after water egress. Furthermore, some diving operations use 100% oxygen to prevent nitrogen narcosis or to mitigate decompression sickness. The increased partial pressure of oxygen (hyperoxia) may exacerbate reductions to human performance (Florian, 2010; Florian et al., 2014; Myers et al., 2019).

With previous hyperoxic exposures, Myers et al. (2019) demonstrated neuromuscular strength performance is significantly altered. Maximum handgrip force dropped 7.8% after the five consecutive, 6-h hyperoxic exposures at 1.35 atmospheres absolute (ATA) (Myers et al., 2019). Additionally, maximal isokinetic (IK) knee extension peak torque dropped by 3.3% (Myers et al., 2019). In both cases, neuromuscular strength performance recovery varied but occurred no later than 72-h post-WI (Myers et al., 2019).

Additionally, these types of exposures are shown to reduce treadmill time-to-fatigue performance by 26% (Florian, 2010). Florian et al. (2011) further demonstrated that time-to-fatigue endurance performance is degraded more after breathing 100% oxygen compared to breathing atmospheric air at 1.35 ATA. Similar hyperoxic exposures at 1.35 ATA in a hyperbaric chamber had comparable effects on treadmill time-to-fatigue endurance performance with a 38% reduction in endurance performance after the fifth exposure (Florian et al., 2012). Recently, Bergeron and Florian (2018) demonstrated that performance remains 31% lower than baseline on 72-h post-WI. Contrarily, 40% maximum handgrip endurance (MHE) time-to-fatigue performance was not affected by the resting hyperoxic exposures indicating that neuromuscular endurance performance in the forearm is unaffected (Florian, 2010; Florian et al., 2011, 2012).

Previous long-duration hyperoxic WIs studies from our laboratory only investigated handgrip endurance (Florian, 2010; Florian et al., 2011, 2012). The previous research did not address load-bearing neuromuscular endurance and associated skeletal muscle tissue oxygenation and neuromuscular activation. As such, the aim of this investigation was to examine the effects of hyperoxic WIs on neuromuscular endurance performance of load-bearing versus non-load bearing muscles, as well as tissue oxygenation. After resting, consecutive, long-duration hyperoxic WIs at 1.35 ATA, we assessed MHE time-to-fatigue and 50-repetition IK knee extension peak torque, neuromuscular activation, and localized tissue oxygenation in attempts to elucidate potential mechanisms of the decrements described above. We hypothesized that over a 5-day period of these exposures, a decrease to neuromuscular endurance performance and tissue oxygenation with the quadriceps muscle would occur but not with the forearm flexors.

## Materials and Methods

### Subjects

Thirteen (*n* = 13) well-trained male divers (V̇O_2max_ = 52.5 ± 5.0 mL/kg/min, mean ± SEM) with an average age of 32 ± 2 years old and 7 ± 5 years (mean ± *SD*) of diving experience participated in this study (Table 1). Prior to beginning the study, each subject underwent a health screening that included a general physical, electrocardiogram, complete metabolic panel, lipid profile, and blood pressure measurements. Exclusion criteria included any known pulmonary, cardiovascular, or metabolic diseases; alcoholism; asthma; and tobacco use. Subjects did not consume any prescription, over-the-counter drugs, or supplements for the duration of the study unless authorized by on-staff medical doctor. A medical doctor, trained in diving physiology, reviewed all subjects’ medical histories. The Institutional Review Boards for the Navy Experimental Diving Unit and Florida State University gave approval for this study. Prior to beginning the study, each subject provided written informed consent, and all procedures conformed to the Declaration of Helsinki.

**TABLE 1:** Subject characteristics.

**Subject demographics**	
*n*	13
Age (year)	31.3 ± 1.7
Height (cm)	177.7 ± 1.8
Weight (kg)	81.4 ± 2.8
Body mass index (kg/m^2^)	25.7 ± 1.7
V̇O_2max_ (mL/kg/min)	52.5 ± 5.0

*Across subject (*n* = 13) (mean ± SEM) characteristics.*

### Study Design

#### Overview

Each subject abstained from food and drink, except water, for 2 h prior to each laboratory visit. Additionally, the subjects were prohibited from performing any fatiguing exercise 48 h prior to any scheduled testing. During all testing sessions, subjects wore running shorts and T-shirts. Subjects completed five consecutive days of 6-h resting WIs with 18-h surface intervals (dive week) with follow-up physiological testing occurring 24 and 72 h after the fifth WI. Physiological testing during the dive week occurred immediately before, and after, each WI. Subjects performed all testing in a climate-controlled laboratory (21.1–22.8°C). Each subject consumed a small, standardized breakfast consisting of 69% carbohydrates, 19% fats, and 12% proteins (350 kcal) and 236.5 mL of orange juice after the pre-WI physiological testing. Before entering the test pool, each subject voided his bladder, was weighed, and donned a condom catheter (Myers et al., 2018b). Subjects breathed 100% oxygen at 1.35 ATA while resting in a reclined position and did not perform any type of physical activity during each 6-h WI. After the WI, each subject removed his catheter, voided his bladder, dried off, and was weighed (Florian et al., 2014; Myers et al., 2018b). A medical doctor specializing in diving physiology was on site during each WI, and evaluated each subject as needed after each WI for potential side effects. Post-WI physiological testing began immediately after weighing and evaluation by the medical doctor. No adverse effects were reported.

#### Familiarization and Baseline Testing

All subjects completed two familiarization sessions no later than 72 h prior to the first WI (Myers et al., 2018b). During these visits, subjects practiced the physiological testing protocols. Dynamometer configuration and sensor-placement measurements were recorded during the first visit to ensure replication during subsequent visits (Myers et al., 2018b).

#### Water Immersion

**FIGURE 1:**
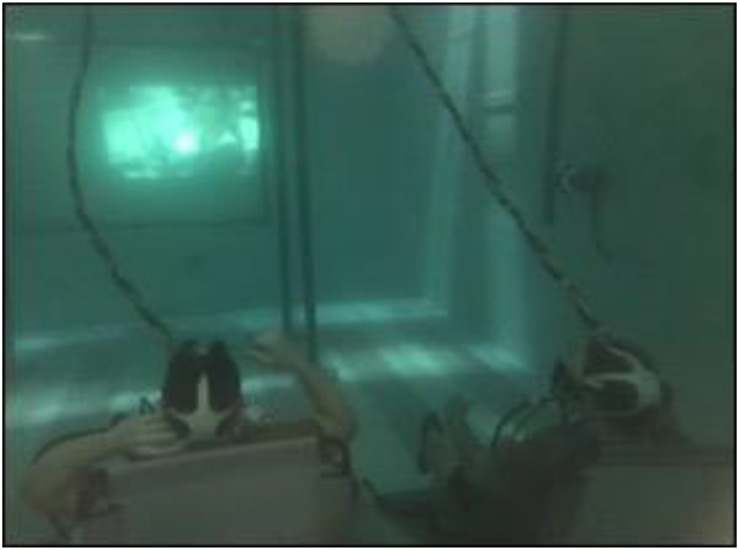
Photo of resting water immersions. Subjects sat in reclining chairs in a thermoneutral (31.7–32.7°C) water tank (15-feet) for 6 h. 100% oxygen was fed from the surface via hoses to the subjects.

The WI protocol occurred as outlined in Florian et al. (2013). Subjects sat in reclining chairs in a thermoneutral (31.7–32.7°C) water tank (15-feet) for 6 h (Figure 1). At the 3-h mark, subjects surfaced up to mid-chest for a 10-min break for a standardized lunch which consisted of 64% carbohydrates, 24% fat, and 12% protein (475 Cal) and 500 mL of liquid (Myers et al., 2018b). Once finished, each subject returned to the bottom of the tank to complete the WI (Florian et al., 2013; Myers et al., 2018b).

### Data Collection

#### Biodex System Pro. 4

Subjects performed the 50-repetition maximal IK extension endurance protocol on a Biodex System 4 Pro. (Biodex Medical Systems, Shirley, NY, United States). The chair angle was maintained at 85° for upper and lower body performance testing (Myers et al., 2018b). Subjects remained seated and strapped to the Biodex during all testing and recovery periods. Subjects did not grasp the Biodex chair or the straps nor were given any verbal encouragement during the exercise protocols. All Biodex attachment measurements and chair configuration measurements were determined during familiarization testing and used throughout the entire study.

#### Endurance Handgrip Equipment

All MHE measurements were taken with the Baseline BIMS HG dynamometer (White Plains, NY, United States) connected to a National Instruments NI USB 6210 module (Austin, TX, United States) and Panasonic Toughbook (Newark, NJ, United States) with National Instruments Labview collection software (Austin, TX, United States) (Myers et al., 2018b). While performing the MHE protocol, each subject flexed his elbow at a 90° angle, wrist straight, and thumb orientated toward the ceiling while gripping the dynamometer. Each subject performed the MHE protocol while sitting on the Biodex. Subjects were not given any type of encouragement during the testing (Myers et al., 2018b).

#### Surface Electromyography (SEMG)

One SEMG (Delsys Trigno Wireless EMG Systems, Boston, MA, United States) sensor recorded neuromuscular activity during the MHE protocol. Each subject’s skin was prepared with an alcohol pad and shaved to ensure proper electrical conductance. A specialized, double-adhesive tape (Delsys Trigno Adhesive, Boston, MA, United States) attached the sensor to the belly of the brachioradialis. All SEMG signals were pre-amplified (100×), amplified (2×), band-pass filtered (10–1,000 Hz), and sampled at 2,000 Hz with Trigno EMGWorks software (version 4.1.7, Boston, MA, United States). Raw SEMG data were converted by using Fourier Transformation root mean squared (RMS) script via EMG Works Analysis software (Delsys, Boston, MA, United States). The raw SEMG amplitude data for all previously mentioned exercise protocols were normalized to baseline and used for statistical analysis (Myers et al., 2018b).

#### Near-Infrared Spectroscopy (NIRS)

This study used a two-wavelength, portable, continuous-wave NIRS system (Portamon, Artinis, Medical Systems, Zetten, Netherlands) to measure oxygen kinetics. The system utilized the modified Beer–Lambert and spatially resolved spectroscopy methods to measure changes in deoxyhemoglobin [HHb] and oxyhemoglobin [HbO_2_] concentrations at 760 and 850 nm wavelengths (Ufland et al., 2012; Myers et al., 2018b). Due to an overlap in wavelengths, the analysis software cannot separate myoglobin concentrations from hemoglobin concentrations. Each NIRS device had three fixed optode sets capable of penetrating 35 mm into the target tissue. A Portamon sensor was placed on the belly of the vastus lateralis and medial forearm flexor muscles, primarily the flexor carpi radialis (Ufland et al., 2012; Myers et al., 2018b). Each placement was measured, marked with indelible ink, and recorded during baseline testing to ensure placement consistency before each test. Previous studies have shown NIRS testing in the same location does not affect the light values (Myers et al., 2018a,b). Black Kinesio-tape attached the probe to the skin to prevent corruption of the signal from ambient light from the surrounding environment (Myers et al., 2018a,b). Skinfold thickness at the sight of probe application was taken using Lange skinfold calipers (Cambridge Scientific Industries, Cambridge, MA, United States) (Myers et al., 2018b). The calculated thickness of the skin and subcutaneous tissue was less than half the distance between the probe and measured target (∼17 mm) (Jammes et al., 2003) (Myers et al., 2018a,b). All NIRS testing was acquired at 10 Hz via a laptop with Windows 8 and recorded for post-exercise analysis (Myers et al., 2018b). HHb concentration and tissue saturation index (TSI) measurements were recorded and analyzed via statistical analysis (Myers et al., 2018b).

### Experimental Procedures

#### MHE Protocol

Maximal handgrip endurance testing occurred pre- and post-WI for the dive week, and 24-h and 72-h post-WI 5. Using a visual force feedback system, subjects held 40 ± 2% of baseline MHG with the right hand until fatigue. A digital real-time output visually dictated whether the subject needed to tighten or lessen his grip to maintain the proper force output. The subject’s time was recorded using a stopwatch and began when the subjects’ grip reached predetermined target. The protocol ended once the subject could no longer maintain the required force or voluntarily discontinued the test (Myers et al., 2018b). The total grip time (seconds) was recorded and analyzed.

Simultaneously, real-time SEMG and NIRS data were recorded. Brachioradialis SEMG data were transformed via RMS and normalized to baseline values. Since time-to-fatigue differed from subject to subject and individual testing sessions, the SEMG normalized amplitude data, HHb values, and TSI values were averaged into quartiles using a proprietary script for MatLab (Version R2016A, The Mathworks, Natick, MA, United States). The results of the quartile analysis were used for statistical analysis (Myers et al., 2018b).

#### 50-Repetition Maximal IK Extension Protocol

The 50-repetition maximal isokinetic knee extension occurred pre- and post-WI 1 and 3, pre-WI 5, and 24-h post-WI 5. The exercise protocol was limited to these days to minimize test-related fatigue. Before beginning each test, the subject was briefed on the exercise protocol and instructed to give maximal effort. Each subject performed one set of 50 repetitions of maximal IK right knee extension at an angular rate of 180°/s concentric and 300°/s eccentric (Myers et al., 2018b) with his right leg. Only one set of the 50-repetition maximal IK knee extension exercise was performed during specified testing sessions as to minimize the effects of fatigue and skeletal muscle microtrauma (Myers et al., 2018b). The peak torque (Newton-meters) for repetitions 2–4, 24–26, and 48–50 and total work were recorded (Myers et al., 2018b). Upon completion of the fatigue protocol, the subject was permitted to rest in place until all NIRS readings returned to pre-exercise levels (Myers et al., 2018b).

Rate of fatigue was calculated as the slope of decline in the concentric peak force outputs. The total work and the rate of fatigue were the variables analyzed (Myers et al., 2018b). NIRS-derived peak and steady-state HHb and TSI values were measured and consolidated via a proprietary script for MatLab version R2016A (Myers et al., 2018b). The relative changes for HHb and TSI were calculated and used for statistical analysis (Myers et al., 2018b).

#### Muscle Oxidative Capacity

The Portamon NIRS sensor placement was used to assess muscle oxidative capacity as outlined in Ryan et al. (2012). A key assumption made about this technique is the signal changes in hemoglobin are proportional to mitochondrial oxygen consumption (Ryan et al., 2013). Three consecutive tests were conducted on the right vastus lateralis with 3 min of rest between each test. A pneumatic cuff was strapped to the most proximal portion of the right leg. The cuff was attached to a pneumatic pump capable of inflating the cuff to suprasystolic pressure equal to 300 mmHg (Myers et al., 2018b). The pneumatic pump was capable of inflating and deflating the cuff within 1 s (Myers et al., 2018b).

**FIGURE 2:**
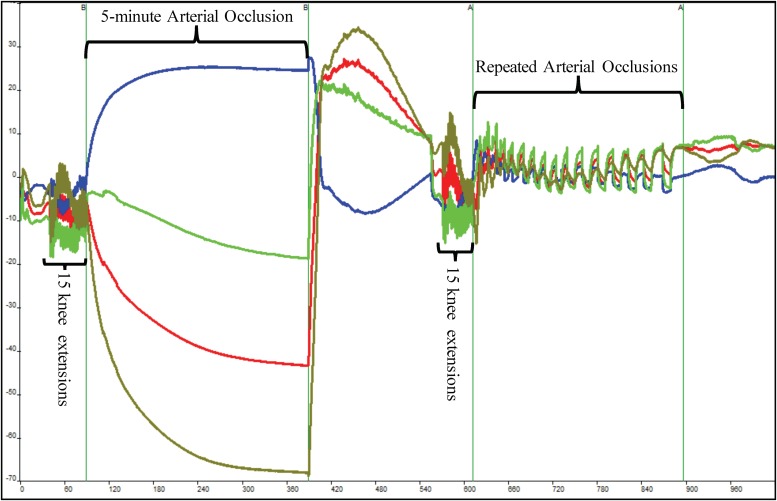
Depiction of raw near-infrared spectroscopy (NIRS) data during a single muscle oxidative capacity (MOC) test. Fifteen IK knee extensions were used to increase skeletal muscle metabolic rate that was followed by a 5-min occlusion induced by a high-pressure cuff. Following a 3-min recovery period, another set of 15 IK knee extensions followed by a series of short arterial occlusions were performed. The blue line indicates deoxyhemoglobin (HHb), red indicates oxyhemoglobin, olive line indicates hemoglobin difference, and green line depicts total hemoglobin.

The subject sat still in the Biodex System Pro. 4 chair for 60 s, after which the pneumatic cuff was inflated and deflated for 30 s. A series of three 10-s occlusions occurred. After the 3-min recovery period, the subject completed 15 IK knee extensions on the Biodex at an angular rate of 300°/s for the concentric and eccentric motions. After the 15th extension, the subject’s right leg was extended to 120°, locked in place using the Biodex leg extension attachment, and the cuff was inflated. The occlusion lasted for 5 min. Once the cuff was released, the subject recovered for 3 min without moving as shown in Figure 2. This protocol served as the 100% and 0% oxygenation HHb levels for the muscle oxidative capacity calculations (Ryan et al., 2012; Myers et al., 2018b).

Following another 3-min recovery period, the subject performed another set of 15 IK knee extensions at 300°/s. Immediately after the 15th extension, the subject’s right leg was extended to approximately 120° and repeated short arterial occlusions were performed. These were used to assess changes in metabolic rate (Ryan et al., 2013). The initial occlusions were 5 s in length followed by 5 s of rest (occlusions 1–5). The subsequent occlusions were 7 s in length followed by 7 s of rest (occlusions 6–10) and 10 s in length and 10 s of rest (occlusions 11–15) as shown in Figure 2. The Portamon NIRS signals were corrected and used to calculate the subject’s muscle oxidative capacity time constant (Tc) and rate constant (k) using MatLab (Mathworks, Natick, MA, United States) as outline in Ryan et al. (2012, 2013).

### Data Analysis

The SPSS Faculty Pack (version 23, IBM, ON, Canada) was used for all statistical analyses with the level of significance set at *p* < 0.05. Data are reported in means ± SEM. For the MHE analysis, a two-way repeated-measures analysis of variance (ANOVA) 2 × 3 (pre-/post-WI × day) model for the dive week was conducted (Myers et al., 2018b). For the muscle oxidative capacity analysis, a two-way repeated-measures ANOVA 2 × 5 (pre-/post-WI × day) model for the dive week was used. MHE and MOC recovery periods were analyzed via a one-way repeated-measures ANOVA (1 × 3) (day) model on pre-WI 1 (baseline), 24-h post-WI, and 72-h post-WI (Myers et al., 2018b). Conversely, for 50-repetition maximal IK knee extension analysis, a two-way repeated-measures ANOVA (2 × 2) (pre-/ post-WI × day) model on WIs 1 and 3 were used (Myers et al., 2018b). Recovery analysis used a one-way repeated-measures ANOVA 1 × 3 (day) model on pre-WI 1 (baseline), pre-WI 5, and 24-h post-WI (Myers et al., 2018b). When appropriate, Bonferroni correction was used to adjust the alpha when *post hoc* tests were performed (Myers et al., 2018b).

## Results

### 40% Maximal Handgrip Endurance (MHE)

As shown in Figure 3, a day main effect occurred with MHE time-to-fatigue (*p* = 0.001). Time-to-fatigue performance dropped by 15% at WI 3 (*p* = 0.009) and by 17% at WI 5 (*p* = 0.002). Performance remained depressed by 22% on 24-h post-WI (*p* < 0.001) and by 12% at 72-h post-WI (*p* = 0.019). As shown in Tables 2, 3 and Figure 4, no changes were observed in SEMG or NIRS measurements.

**FIGURE 3:**
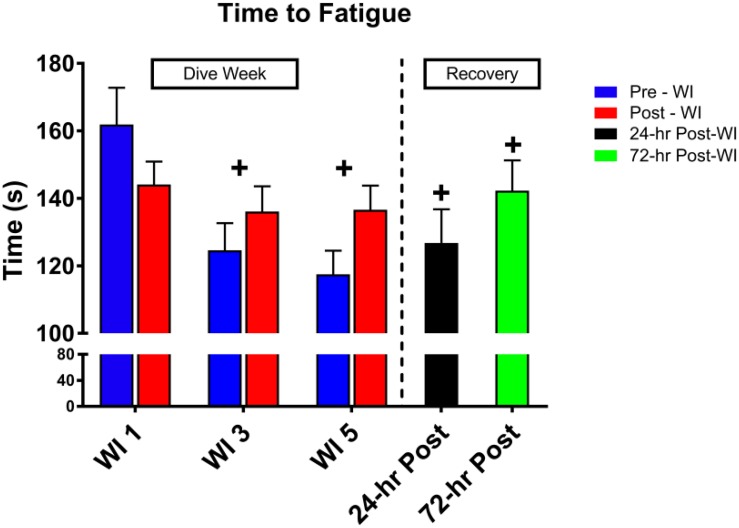
Forty percent maximum handgrip endurance (MHE) time-to-fatigue results. Across subject (*n* = 13) mean ± SEM is shown. Time-to-fatigue times dropped on WI 3 and did not recover by 72-h post-WI. “+” signifies day main effect (*p* < 0.05).

**TABLE 2:** Forty percent maximum handgrip endurance (MHE) surface electromyography (SEMG) results.

	**Time**					***p*-value**			
	**Dive day**			**Recovery**		**Main effect**		**Interaction**	**Main effect**
	**Day 1**	**Day 3**	**Day 5**	**24-h post**	**72-h post**	**Pre/post**	**Day**	**Pre/post × day**	**Recovery**
**SEMG, normalized RMS (%)**									
Pre	100 ± 0	121 ± 6	107 ± 10	120 ± 17	112 ± 15	0.682	0.993	0.372	0.731
Post	103 ± 7	117 ± 11	113 ± 9	–	–				
**SEMG, normalized RMS (%) – Quartile 1**									
Pre	100 ± 0	112 ± 13	103 ± 7	108 ± 15	111 ± 13	0.278	0.243	0.247	0.678
Post	105 ± 9	117 ± 20	102 ± 9	–	–				
**SEMG, normalized RMS (%) – Quartile 2**									
Pre	100 ± 0	108 ± 15	109 ± 14	116 ± 19	111 ± 18	0.222	0.215	0.367	0.647
Post	99 ± 12	101 ± 18	96 ± 13	–	–				
**SEMG, normalized RMS (%) – Quartile 3**									
Pre	100 ± 0	109 ± 14	109 ± 11	137 ± 28	130 ± 33	0.272	0.135	0.247	0.358
Post	109 ± 13	104 ± 23	105 ± 15	–	–				
**SEMG, normalized RMS (%) – Quartile 4**									
Pre	100 ± 0	100 ± 11	122 ± 16	126 ± 31	115 ± 15	0.345	0.117	0.091	0.302
Post	120 ± 24	96 ± 19	89 ± 7	–	–				

*All results (*n* = 13) reported as mean ± SEM.*

**TABLE 3:** Forty percent MHE total saturation index (TSI) results.

	**Time**					***p*-value**			
	**Dive day**			**Recovery**		**Main effect**		**Interaction**	**Main effect**
	**Day 1**	**Day 3**	**Day 5**	**24-h post**	**72-h post**	**Pre/post**	**Day**	**Pre/post × day**	**Recovery**
**TSI (%)**									
Pre	62.51 ± 1.81	62.11 ± 0.99	63.43 ± 1.09	60.22 ± 1.02	60.96 ± 1.27	0.702	0.939	0.757	0.923
Post	61.77 ± 4.79	63.57 ± 4.21	60.4 ± 1.69	–	–				
**TSI (%) – Quartile 1**									
Pre	62.34 ± 1.39	62.22 ± 0.73	63.35 ± 0.94	61.37 ± 0.8	60.91 ± 1.54	0.862	0.921	0.761	0.314
Post	62.51 ± 4.84	64.68 ± 3.78	61.94 ± 1.77	–	–				
**TSI (%) – Quartile 2**									
Pre	62.53 ± 1.82	61.68 ± 0.95	62.8 ± 1.31	59.99 ± 1.28	61.32 ± 1.39	0.653	0.998	0.906	0.057
Post	61.23 ± 5.03	62.25 ± 3.76	60.8 ± 1.57	–	–				
**TSI (%) – Quartile 3**									
Pre	63.04 ± 1.95	62.62 ± 1.21	64.09 ± 1.61	59.98 ± 1.28	61.33 ± 1.38	0.37	0.902	0.701	0.108
Post	61.33 ± 4.52	63.35 ± 4.39	59.51 ± 1.84	–	–				
**TSI (%) – Quartile 4**									
Pre	62.15 ± 2.36	61.97 ± 1.48	63.57 ± 1.35	59.68 ± 1.36	60.99 ± 0.74	0.705	0.877	0.658	0.125
Post	62.02 ± 4.91	63.99 ± 5.07	59.36 ± 1.91	–	–				

*All results (*n* = 13) reported as mean ± SEM.*

**FIGURE 4:**
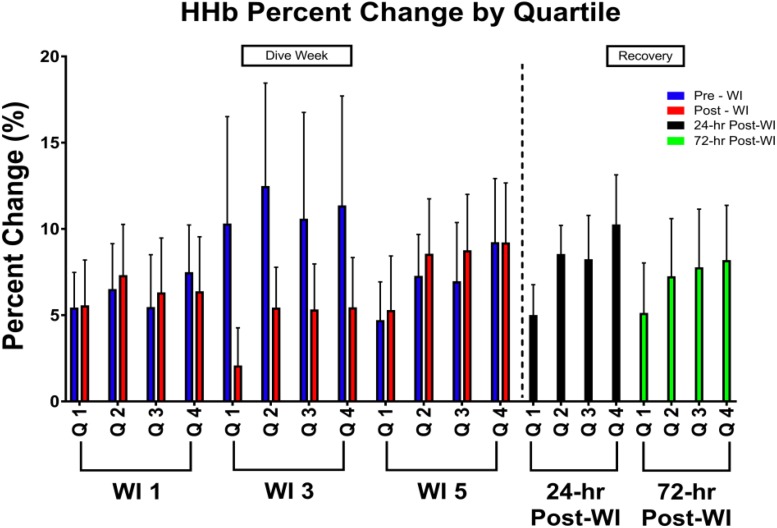
Forty percent MHE HHb percent change by quartile results. Across subject (*n* = 13) mean ± SEM is shown. Each WI quartile was compared to its baseline counterpart. For example, WI 3 Q1 compared to WI 1 Q1; WI 3 Q2 compared to WI 1 Q2; WI 5 Q1 compared to WI 1 Q1; WI 5 Q2 compared to WI 1 Q2. No significance was observed.

### 50-Repetition Maximal IK Knee Extension

**FIGURE 5:**
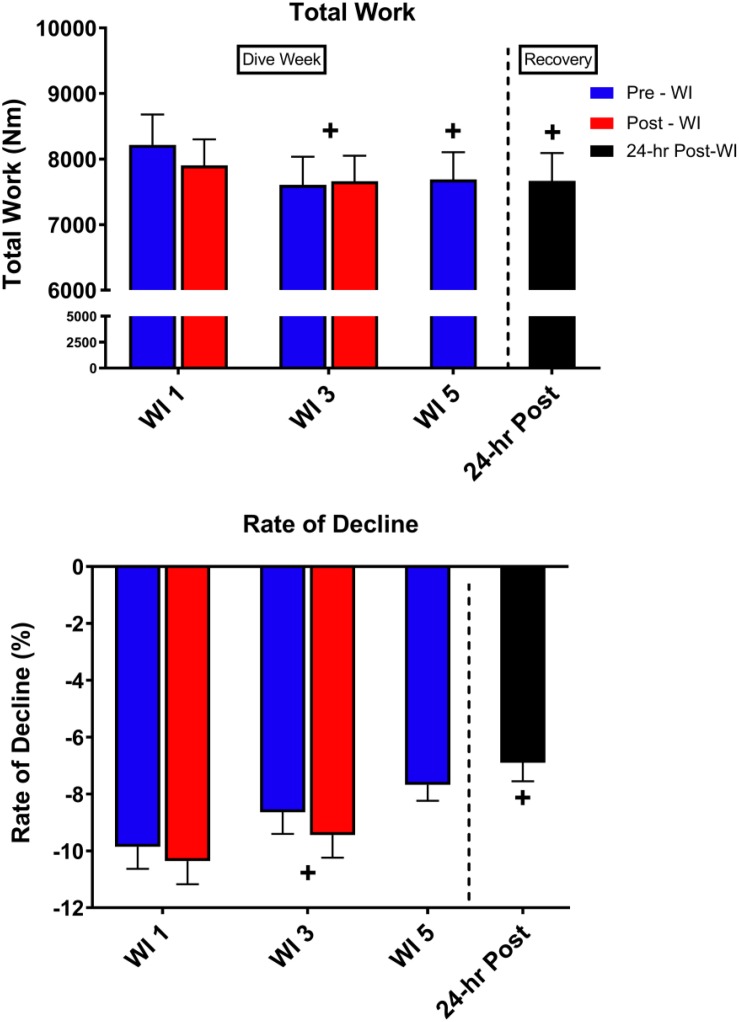
Fifty-repetition maximal IK knee extension performance results. Across subject (*n* = 13) mean ± SEM is shown. Total work was lowest on WI 3. However, the rate of fatigue was greatest on WI 1. “+” signifies day main effect (*p* < 0.05).

As shown in Figure 5, total work decreased by 5% (*p* = 0.002) on WI 3 with a 7.5% decline at pre-WI 5 and 12.3% (*p* = 0.032) decrease at 24-h post-WI. A *post hoc* comparison of the force outputs of repetitions 2–4 for post-WI 1 and pre-WI 3 showed post-WI 1 was 9% greater (*p* = 0.018). The rate of fatigue was 12.4% (*p* = 0.028) greater at WI 1 than at WI 3, and 30% greater (*p* = 0.017) WI 1 than on 24-h post-WI.

**FIGURE 6:**
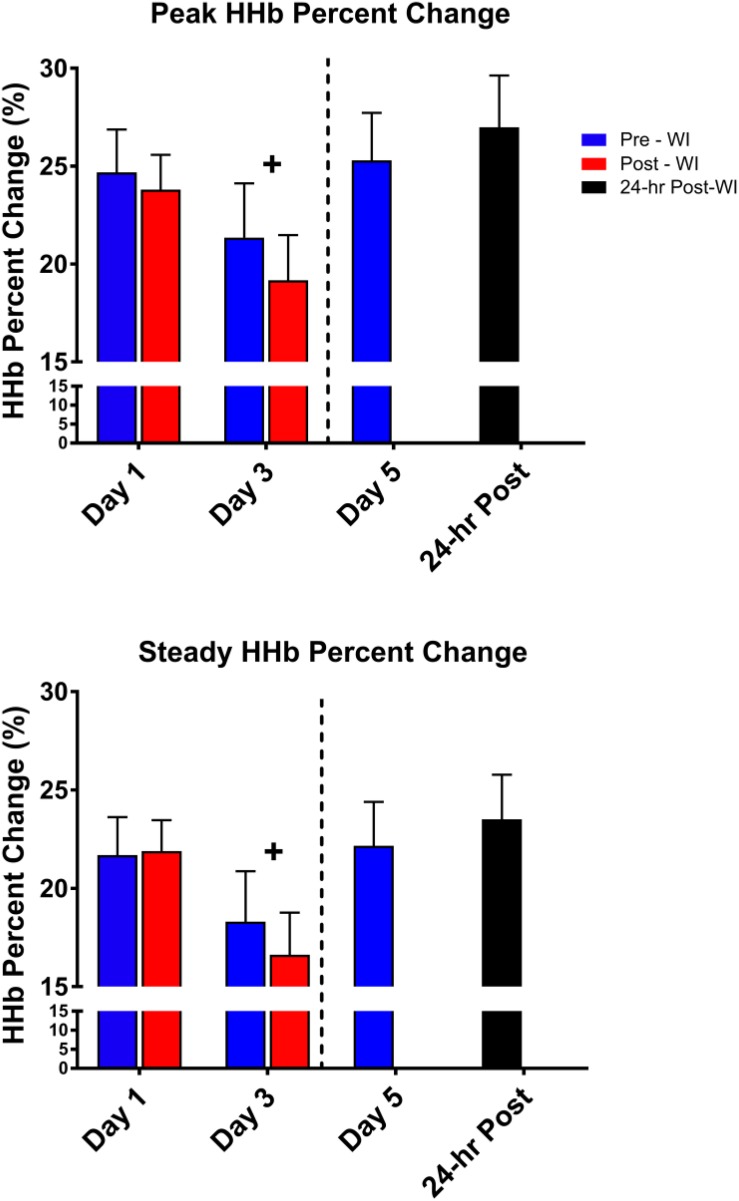
Fifty-repetition maximal IK knee extension NIRS results. Across subject (*n* = 13) mean ± SEM is shown. HHb concentrations were lowest on WI 3. “+” signifies day main effect (*p* < 0.05).

As shown in Figure 6, peak HHb concentrations dropped by 17% (*p* = 0.04) and steady-state HHb dropped by 20% (*p* = 0.016) at WI 3 when compared to WI 1. For both HHb metrics, pre-WI 5 and 24-h post-WI measurements were similar to WI 1 measurements. The TSI peak and steady-state metrics did not have a statistically significant day main effect as shown in Table 4.

**TABLE 4:** Fifty-repetition IK knee extension total saturation index (TSI) quartile results.

	**Day 1**	**Day 3**	**Day 5**	**24-h post**	**72-h post**	**Pre/post**	**Day**	**Pre/post × day**	**Recovery**
**TSI peak value (%)**									
Pre	53.51 ± 1.43	53.74 ± 1.57	51.04 ± 1.56	53.45 ± 1.41	–	0.687	0.980	0.977	0.152
Post	54.24 ± 1.57	53.94 ± 2.27	–	–	–				
**TSI steady state value (%)**									
Pre	58.32 ± 1.06	57.45 ± 1.29	55.81 ± 1.46	57.75 ± 1.29	–	0.407	0.889	0.798	0.164
Post	57.45 ± 1.29	57.36 ± 1.61	–	–	–				

*All results (*n* = 13) reported as mean ± SEM.*

### Muscle Oxidative Capacity (MOC)

No changes were observed with MOC measurements as shown in Table 5.

**TABLE 5:** Muscle oxidative capacity (MOC) results.

	**Time**							***p*-value**			
	**Dive day**					**Recovery**		**Main effect**		**Interaction**	**Main effect**
	**Day 1**	**Day 2**	**Day 3**	**Day 4**	**Day 5**	**24-h post**	**72-h post**	**Pre/post**	**Day**	**Pre/post × day**	**Recovery**
**k**											
Pre	1.81 ± 0.15	2.11 ± 0.18	2.07 ± 0.25	2.26 ± 0.12	1.96 ± 0.12	2.22 ± 0.19	1.98 ± 0.18	0.869	0.151	0.243	0.338
Post	2.29 ± 0.26	2.21 ± 0.15	1.9 ± 0.13	1.9 ± 0.13	1.91 ± 0.25	–	–				
**Tc**											
Pre	36.92 ± 2.74	31.46 ± 2.94	36.84 ± 5.96	31.06 ± 3.03	32.03 ± 2.45	28.62 ± 2.43	35.56 ± 2.43	0.986	0.26	0.19	0.059
Post	33.0 ± 2.91	28.38 ± 2.64	26.5 ± 1.93	33.27 ± 2.39	36.45 ± 4.98	–	–				

*All results (*n* = 13) reported as mean ± SEM.*

## Discussion

The purpose of this study was to determine the extent to which consecutive, resting long-duration 100% oxygen WIs would affect neuromuscular endurance performance and recovery. We hypothesized that load bearing neuromuscular endurance performance would be reduced following repeated long-duration hyperoxic exposures with increased recovery time. Our findings confirm the central hypothesis for this study. The data show significant decreases in the 50-repetition IK knee extension and MHE performance results. The decrease in MHE time-to-fatigue results was contrary to our hypothesis.

### MHE Performance

We hypothesized that these hyperoxic WIs would not change MHE performance due to the historical data (Florian, 2010). Previously, Florian (2010) had shown no changes in MHE performance. In this study, we demonstrated a 17% drop in time-to-fatigue performance by WI 5 with a residual decline in performance at 72-h post-WI by 12% as seen in Figure 3. The difference between the Florian et al. MHE protocol and this study’s MHE protocol is the way each subject performed the handgrip position.

In the Florian et al. studies, subjects were in the supine position with his arm outstretched to the side (Florian, 2010). In the current study, subjects sat upright with their elbow flexed 90° with the humerus parallel to the floor (Myers et al., 2017, 2018a). The subject’s posture can influence the outcome of the test, particularly for strength (El-Sais and Mohammad, 2014). However, Shyam Kumar et al. (2008) showed elbow position does not cause changes to grip endurance testing results. El-Sais and Mohammad (2014) demonstrated that prone maximum handgrip strengths were lower than those of the seated position. The El-Sais and Mohammad (2014) study did not investigate endurance performance, but the maximum handgrip strength differences in body position may explain the differences between our MHE results to those of Florian et al. (Florian, 2010).

Despite the differences in grip positions, the results of this study show repeated hyperoxic WIs cause significant decreases in MHE time-to-fatigue performance. Previously, we illustrated that similar 6-h normoxic WIs in the seated position do not cause decrements to MHE time-to-fatigue performance (Myers et al., 2017). The presence of two differing inspired gases is the most likely causalities of this difference in results between the two studies.

### 50-Repetition IK Knee Extension Performance

As shown in Figure 5, total work dropped by 5% on WI 3 and continued to drop by 12.3% on 24-h post-WI. With this drop in performance, one would expect the rate of fatigue to be greater on WI 3. However, this was not the case. The rate of fatigue was 10% greater at WI 1 than at WI 3 and 30% greater than 24-h post-WI. One possible explanation for the variances in total work and rate-of-fatigue is the difference in the force outputs during the initial IK repetitions.

We found differences in the initial repetitions. For this study, we used repetitions 2–4, 24–26, and 48–50 to calculate the rate-of-fatigue for this exercise protocol. *Post hoc* analysis guided by a visual analysis of Figure 5 illustrated a significant difference between post-WI 1 and pre-WI 3. A paired *t*-test of the force outputs of repetitions 2–4 for post-WI 1 and pre-WI 3 showed post-WI 1 was 9% greater (*p* = 0.018) than pre-WI 3. In addition, pre-WI 5 was 7.5% lower than pre-WI 1. This result demonstrates how after one bout of hyperoxic WI exposure reduces muscular endurance performance and continues to cause muscular fatigue. The differences in rate of fatigue on WIs 1 and 3 further support this conclusion.

The average peak torque for repetitions 2–4 at pre-WI were 117.2 nM; the peak torque for repetitions 2–4 on pre-WI 3 were 108.4 nM. This difference in initial peak torque shows the first three repetitions at pre-WI 3 were 7.5% lower than pre-WI 1. This means that the slope or rate of fatigue at pre-WI 3 is less than on pre-WI 1. This pattern shows that the muscle was already fatigued. Once fatigued, the rate of fatigue for WI 3 will be less than WI 1. The decrease in the initial repetitions and total work throughout the dive week demonstrates the progression of fatigue produced by the hyperoxic exposures. We can definitively show the hyperoxic exposures are the causality when we compare these results to our previously published research (Myers et al., 2018b).

### MOC and Oxygen Off-Loading Kinetics

Oxygen availability was not a limiting factor for the decreases in neuromuscular endurance performance. The NIRS measurements from this study support this statement. Foremost, no change occurred in the MOC measurements as shown in Table 5. The data suggest no changes occurred to mitochondrial function; the mitochondrial capacity for energy production was unaltered. This result needs to be interpreted with caution. The reductions in neuromuscular endurance performance strongly suggest an impairment to aerobic respiration and mitochondrial capacity. These possible mitochondrial changes may be smaller than the sensitivity of the MOC testing can detect. Furthermore, mitochondrial testing is necessary to fully understand the possible mechanisms involved.

No changes in HHb concentrations or TSI occurred during the MHE testing. Additionally, HHb concentrations decreased during the 50-repetition IK knee extension protocol during the dive week with no statistically significant changes in TSI (Table 3). These metrics suggest oxygen availability was not a limiting factor during the exercises. These results support the conclusion that hyperoxia does not affect oxygen offloading kinetics or mitochondrial function.

### Hyperoxia and Neuromuscular Endurance

This study shows consecutive hyperoxic WIs cause decrements to performance metrics and prolong recovery. These changes to performance and recovery are not seen with normoxic dives of this same length and pressure (Myers et al., 2018b). However, there are similarities between patterns of neuromuscular endurance in this study and aerobic endurance previously reported. Florian et al. (2011) showed treadmill time-to-fatigue endurance performance is reduced by 34% immediately following five consecutive, long-duration, WIs, and hyperbaric chamber exposures with divers breathing to 100% oxygen at 1.35 ATA. Our data show similar declines.

Even though MHE and 50-repetition IK knee extension protocols are not purely aerobic in nature, they share an aerobic component. The length of time for these exercises lasted between 2 and 5 min. Powers and Howley (2011) state that about 60% of adenosine triphosphate is produced through aerobic processes with efforts falling into this effort and time range. This shared aerobic process between the current neuromuscular endurance results and Florian et al.’s time-to-fatigue results partially explains the similar decrements between the results.

If we combine the results from all the previously mentioned hyperoxic WIs studies, a pattern emerges. The effects of hyperoxic WIs on neuromuscular strength by Myers et al. (2018b) demonstrates neuromuscular maximal strength performance decreases about 5% after three consecutive exposures with continuing decrements lasting beyond the 72-h post-WI recovery period. With this study, we show that neuromuscular endurance performance decreases between 5 and 15% after three consecutive exposures with continuing declines in performance lasting beyond the 72-h post-WI recovery period. Finally, the Florian et al. studies show aerobic performance declines by 38% immediately following five consecutive hyperoxic exposures with a continual reduction of performance by 31% at the 72-h post-WI recovery period (Florian et al., 2012; Bergeron and Florian, 2018). The causality of these reductions in performance is not entirely clear. With this study, we showed that no changes occur to indirect measures to mitochondrial capacity or oxygen kinetics. We can infer oxygen availability is not a limiting factor in the aerobic respiration process (Powers and Howley, 2011; Ryan et al., 2012, 2013; Myers et al., 2018b). However, previous studies have shown increased reactive oxygen species generation from hyperoxic exposures.

Brerro-Saby et al. (2010) and Jammes et al. (2003) demonstrated hyperoxic hyperbaric exposures at 1.15 ATA cause immediate increases in reactive oxygen species. Additionally, these studies saw reductions in tonic vibration response neuromuscular amplitude and changes in M-wave characteristics. The authors suggest these results indicate a reduction in resting membrane potential (Jammes et al., 2003; Brerro-Saby et al., 2010). Myers et al. (2019) reported similar decreases in neuromuscular activation during maximal strength exercise performance after hyperoxic exposures at 1.35 ATA. Brerro-Saby et al. (2010) hypothesizes the overproduction in free radicals may cause changes to the skeletal muscle α and γ motor neurons which causes the changes to the neuromuscular activation. Even though we did not see changes to neuromuscular activation during the muscular endurance protocols in this study, changes in neuronal signaling may still be occurring. Any neuronal changes may still play a part in the reduction on endurance performance. Further investigation is required to explore this area.

### Experimental Considerations

This study is the first to consider the effects of consecutive, resting, long-duration hyperoxic WIs on skeletal muscle endurance, neuromuscular activation, and HHb concentrations. Nevertheless, special consideration must occur when interpreting the results of this study. At the outset, the exercise protocols occurred 1–1.5 h after WI. If changes in oxygen offloading kinetics occurred prior to protocol initiation, we could not detect these changes. Additionally, the HHb concentrations were not normalized to ischemic or hyperemic responses (Ryan et al., 2012, 2013). By performing the ischemic protocol as described for the MOC testing procedure, a 0–100% saturation curve is created to normalized HHb values recorded during the exercise protocols. If we were to follow typical ischemic protocols on the forearm and quadriceps during the entire dive week, we would have caused undue stress and discomfort to the subjects (Ryan et al., 2012, 2013). Furthermore, disagreement exists on how to normalize raw NIRS data in the absence of the ischemic protocol (Ufland et al., 2012). It is unclear if this change in procedure would change the results.

The conclusions drawn from the 50-repetition maximal IK knee extension test are limited. The performance data are limited in explaining the both main effects on WIs 1 and 3. Further reduction in performance could have occurred post-WI 5. This protocol was not tested post-WI 5 as to not to interfere with other exercise protocols. More specifically, the 50-repetition maximal IK knee extension protocol would have caused undue fatigue and confounded the results of the other testing.

The MOC protocol is an indirect measurement of mitochondrial capacity through analyzing the rate changes of oxygen consumption of the target muscle. This protocol is well validated in analyzing changes in the mitochondrial capacity. The changes observed in neuromuscular endurance performance in this study could be linked to reductions in the mitochondria’s ability to produce adenosine triphosphate. However, the MOC protocol may not be sensitive enough to detect changes in the target muscle mitochondria. Even though no changes were observed with this particular measurement, more investigation is needed. Future research will need to analyze target tissue mitochondria after these types of hyperoxic exposures to determine if changes in mitochondrial capacity are occurring.

## Conclusion

Our findings demonstrate that repeated 6-h resting, consecutive, hyperoxic WIs specific to this study design cause changes in muscular endurance. The decrements to neuromuscular endurance in this study show reductions to human performance progress due to these types of hyperoxic exposures. No clear mechanism is apparent for these changes in performance. Oxygen availability does not appear to be a limiting factor. Further research is necessary to understand the mechanisms involved in order to find future ways to counter the detrimental effects of hyperoxia on neuromuscular performance.

## Data Availability

The datasets generated for this study can be found in the PRIDE archive, https://www.ebi.ac.uk/pride/archive/, accession number PXD013604.

## Ethics Statement

The Institutional Review Boards for the Navy Experimental Diving Unit and Florida State University gave approval for this study. Prior to beginning the study, each subject provided written informed consent, and all procedures conformed to the Declaration of Helsinki.

## Author Contributions

CM, J-SK, KM, and JF conceived and designed the research study. All authors conducted the research. CM analyzed the data and wrote the manuscript. All authors read and approved the manuscript.

## Conflict of Interest Statement

The authors declare that the research was conducted in the absence of any commercial or financial relationships that could be construed as a potential conflict of interest.
